# Melanotic neuroectodermal tumor of infancy in ovary: A rare case report

**DOI:** 10.1097/MD.0000000000018181

**Published:** 2019-12-10

**Authors:** Zan Liu, Ming Li, Xianglian Tang, Yaling Xiao, Zhenghui Xiao, Yong Li

**Affiliations:** a Department of Pediatric Surgery, Hunan Children's Hospital; b Emergency Center of Hunan Children's Hospital, Changsha, Hunan, China.

**Keywords:** diagnosis, infant, melanotic neuroectodermal tumor of infancy, ovary

## Abstract

**Rationale:**

Melanotic neuroectodermal tumor of infancy (MNTI) is an extremely rare benign pigmented neoplasm of neural crest origin with rapid expansile growth and a high recurrence rate. It is predominantly found in infants of <1 year of age, involvement of the head-and-neck region is the most common presentation though it is reported at other sites including mediastinum, shoulder, thigh, foot, epididymis, uterus and ovary. The patient reported here is the third case of MNTI presenting in an ovary, and the first reported in the infant ovary.

**Patient concerns:**

A 33-month-old girl was presented to our unit for a huge abdominal mass.

**Diagnosis:**

MNTI was eventually diagnosed by histological manifestations supplemented with immunohistochemical findings.

**Interventions:**

Exploratory laparotomy and complete resection were conducted successfully.

**Outcomes:**

Postoperative course was uneventful and no recurrence was displayed in the 6-month follow-up.

**Lessons:**

This case emphasizes that pediatric surgeons and pathologists must always consider the possibility of MNTI while dealing with ovarian neoplasms in infants. Although considered to be a benign tumor, proper treatment and close clinicoradiological follow-up of this tumor are of great importance

## 1 Introduction

Melanotic neuroectodermal tumor of infancy (MNTI) is an extremely rare benign pigmented neoplasm with rapid expansile growth and a high recurrence rate. Since the first description by Krompecher as congenital melanocarcinoma in 1918,^[1]^ approximately 500 cases have been reported in the medical literature till date.^[2]^ Other synonymous terms including melanotic progonoma, congenital pigmented epulis, pigmented adamantinoma, retinal anlage tumor, pigmented papillary medulloblastoma and pigmented tumor of the jaw of infants have also been denominated for this unusual tumor. The differences in terminology reflects the elusive origin of this tumor, which prevailed for half a century. To date, the neural crest cell origin proposed by Borello and Gorlin in 1966 appears to be the most plausible.^[3,4]^

MNTI frequently occurs in infants with no obvious sex predilection in their first year of life,^[5,6]^ 80% of which are less than 6-month-old.^[3]^ Up to 92.8% of this tumor occurred in the head and neck region, especially in the maxilla, followed by skull, mandible and brain.^[3]^ It affected the epididymis, mediastinum, ovary, uterus, thigh, foot and shoulder occasionally.^[3,7]^ Although MNTI is generally considered a benign tumor, it is characterized by rapid growth, certain invasiveness and metastasis, and a high recurrence rate.^[8,9]^ Similar to other tumors of neural origin such as pheochromocytoma, neuroblastoma and retinoblastoma, the urinary excretion of vanillylmandelic acid (VMA), the main end-stage metabolite of catecholamines, was frequently found being elevated in this tumor. However, this symptom alone is not enough to diagnose MNTI.^[5]^

Surgical excision is the preferred treatment of choice for this tumor in most of the cases. Other treatments may be acceptable for those are not amenable to surgical management alone, for example, chemotherapy, chemotherapy with radiotherapy, chemotherapy combined with surgical treatment.^[7]^ Notably, the benefits of radiotherapy in the management of MNTI are unproven yet.^[10]^ Though MNTI is a benign lesion, owing to its relatively high recurrence rate, close follow-up must be conducted during the postresection period.

## 2 Case presentation

### 2.1 General information

A 33-month-old girl, well developed and otherwise healthy, was referred to our department after her parents found abdominal mass for half a month. The infant did not have any symptoms of abdominal pain, vomiting, fever and loss of weight. She was a full-term normal vaginal delivery with birth weight of 3.0 kg, there is nothing unusual during pregnancy and childbirth.

### 2.2 Examination

**Figure 1:**
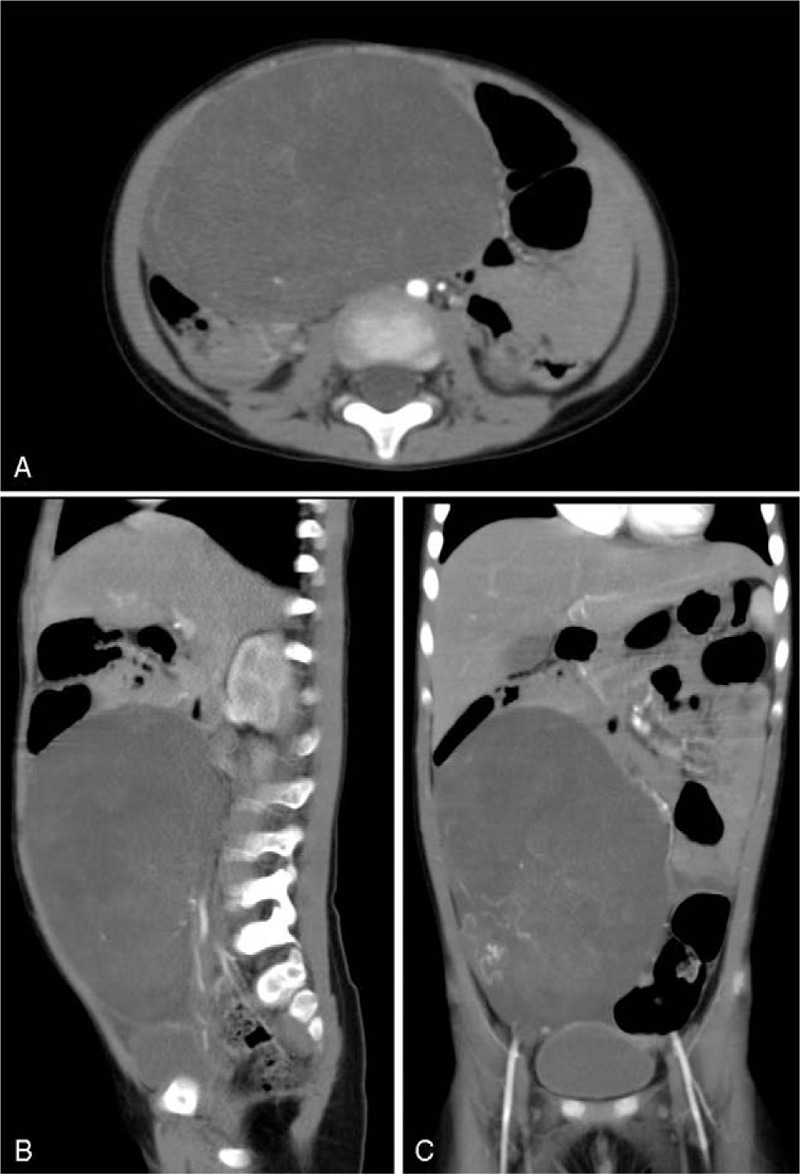
Radiologic features of a melanotic neuroectodermal tumor of infancy in a 33-month-old girl. Computerized tomography scans of horizontal (A), sagittal (B) and coronal (C) plane demonstrating a huge solid mass with no uniform density reached the lower edge of the liver and descended to the entrance of the pelvic cavity, lacking obvious calcification and fat density.

On physical examination, a hard mass with a diameter of about 10 cm on the right abdomen can be palpable, with clear boundary, poor mobility and no tenderness. No other abnormality was found on systemic examination. Except the Lactate dehydrogenase (LD) reached 832 IU/L, routine blood investigation including hemogram, liver function tests, and kidney function tests were within normal limits. In addition, examination of alpha-fetoprotein (AFP), carcinoembryonic antigen (CEA), serum ferritin (SF) and urinary VMA were all not abnormal. The computerized tomography (CT) scans of the abdomen showed a huge solid mass with no uniform density and obvious inhomogeneous enhancement on enhanced scans, measuring 12.6 × 9.0 × 10.1 cm, in the right mid-lower abdomen and part of the pelvic region, the lesion reached the lower edge of the liver and descended to the entrance of the pelvic cavity, lacking obvious calcification and fat density (Fig. 1, A–C).

### 2.3 Operation

At surgery, the huge mass with intact capsule in the abdominal cavity was found to be originated from the right ovary, then complete resection was accomplished successfully. The result of intraoperative frozen section examination suggested it was a small-round cell malignant tumor, so the right adnexa was also extensively resected. The patient's postoperative course was uneventful.

### 2.4 Pathological results

**Figure 2:**
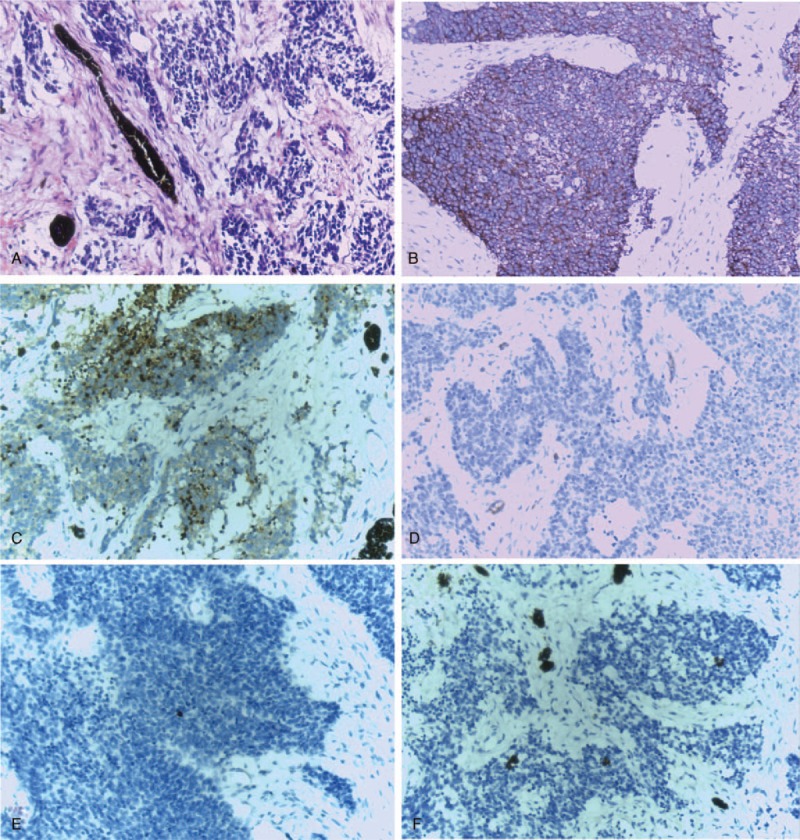
Photomicrographs of histologic and immunohistologic findings in melanotic neuroectodermal tumor of infancy. (A) H&E staining of this ovarian MNTI showing a small round cell tumor, with neuroblast-like cells and scattered melanin particles in tumor areas. IHC analysis indicating positive expression for CD56 (B) and NSE (C), while negative expression for CD99 (D), S-100 (E) and PHOX2B (F). (magnification 200×).

Grossly, the complete excision specimen measured 12.5 × 9.0 × 7.5 cm. Cut surface of the tumor was gray-white, multi-area were fish-like, with visible necrosis. Microscopic examination revealed it was a small round cell tumor, and some small round cells were differentiated into neuroblastoma (Fig. 2A). Nerve fiber filaments and scattered melanin particles were found in tumor areas, and significant hyperplasia of fibrous tissue in interstitial components of tumors. Ovarian interstitial components were observed in small protuberances on the surface of the mass, and no tumor cells were involved. Immunohistochemical analysis demonstrated that CD56 and neuron-specific enolase (NSE) were positive in tumor cells, while CD199, S-100 and PHOX2B were negative (Fig. 2 B–F), and staining for the proliferation marker Ki-67 showed 60% positive cells. According to microscopic and immunohistochemistry findings, a final diagnosis of MNTI was rendered. This case was followed up for 6 months postoperatively without any recurrence.

## 3 Discussion

MNTI is a very rare type of childhood neoplasm. In the past, it was named congenital melanoma, retinal anlage tumor, melanotic progonoma and so on,^[11]^ because of the uncertain origin of tissue. Until 1966, Borello and Gorlin found that preoperative urinary VMA levels were elevated in a 3-month-old male patient, but returned to normal level after surgery,^[3,4]^ suggesting that this tumor might originate from the nerve crest, and suggested the name “melanotic neuroectodermal tumor of infancy ”. Nowadays, it has been confirmed that the tumors originate from the nerve crest, and the nomenclature of MNTI has been approved by the World Health Organization.^[12]^

MNTI was predominant in infants under 1 year of age,^[5,6]^ with an average of 5 months. A few cases have been reported in older children and even adults. MNTI mainly affects the head and neck region, the typical lesion site was the maxilla (68–80%), followed by the skull, mandible and brain.^[3]^ It has also been reported to involve other sites with minimal incidence, such as the femur, epididymis, skull, mediastinum, shoulder, oropharynx, thigh, uterus, and ovary.^[5]^ Noteworthy is that MNTI originated in ovary was extremely rare, we found only 2 reports in which rather similar tumors have been described in the ovary; Hameed et al firstly reported a melanotic neoplasm, that is, MNTI, occurred in the ovary of a 64-year-old woman,^[13]^ and Vajtai et al found the nonepithelial component of MNTI from ovarian mature teratoma (dermoid cyst) of an otherwise healthy 39-year-old woman.^[14]^ However, to the best of our knowledge, an ovarian tumor such as this has never been reported to occur in infants, despite the fact that the overwhelming majority of MNTI affect infants. Additionally, ovarian MNTI supported the concept of developmental versatility of the embryonic neuroectoderm, rather than reflected the origin of tumor cells from organ-specific lineages.^[14]^ In fact, the ovary organ develops from the ectoderm, this histological basis lays a possibility that MNTI occur in the ovary.

Microscopically, MNTI is characterized by two types of cells; one is large cubic epithelioid cells, containing melanin, arranged in alveolar or tubular structures, the other is small neuroblast-like cells, often surrounded by epithelioid cells. Intriguingly, Overwhelming proportions of the neuroblast-like cells in our case made it difficult to differentiate from neuroblastoma. Immunohistochemistry (IHC) markers are useful in differentiating MNTI from various other small cell tumors of infancy, such as embryonal rhabdomyosarcoma, Burkitt lymphoma, neuroblast, malignant neuroectodermal tumors.^[7]^ IHC showed that the large epithelioid/melanocytic cells were positive for cytokeratin (CK), vimentin and HMB-45, and partly for S-100, while the small neuroblast-like cells were positive for S-100, NSE and CD56. Which was basically consistent with this case. Although IHC fails to predict the biological behavior of tumors, Ki-67 and CD99 may suggest that tumors are more aggressive,^[1]^ which reminders us that this patient warrants close follow-up because of high proliferation activity of the tumor cells indicated by ki-67. Recently, Barnes et al found a germline mutation of CDKN2A and a novel RPLP1-C19MC fusion in MNTI,^[9]^ and Gomes et al detected the BRAFV600E oncogenic mutation,^[15]^ suggesting that those genomic, transcriptomic and epigenetic alterations may be applied for diagnosis of MNTI in the near future.

It is difficult to diagnose MNTI preoperatively, and sometimes it is also very tough to differentiate it from other pediatric “small round cell” neoplasms under the microscope.

For those ovarian-derived MNTI, it should be differentiated from the following tumors:

(1) malignant melanoma, with obvious atypia and nuclear fission of tumor cells, easy mitotic image, negative CK and epithelial membrane antigen (EMA) of tumor cells;
(2) embryonic rhabdomyosarcoma, with few pigments in the tumors, often with loose myxoid stromal background, myogenic markers, such as desmin and sarcoplasmic protein expressed in tumor cells, but without CK, HMB45 or NSE;
(3) Metastatic neuroblastoma, with rich nerve fiber reticulum, but few of them contained pigments, tumor cells mainly expressed NSE, PHOX2B, but did not express CK or HMB45;
(4) desmoplastic small round cell tumor (DSRCT), an aggressive small cell neoplasm with distinctive clinical and pathologic features, usually arises in intimate association with the serosal membrane of the peritoneal cavity, harbors a signature translocation t(11;22)(p13,q12).^[16]^

Besides, Ewing sarcoma, rhabdomyosarcoma, peripheral neuroepithelioma, peripheral primitive neuroectodermal tumor, and lymphoma should also be differentially diagnosed with MNTI.^[3]^

The clinical manifestations of this tumor are mainly local expansive or invasive growth, and grow rapidly, but usually benign. Complete surgical resection is the main method of treatment, other treatment includes chemotherapy, radiotherapy and chemotherapy, surgery combined with chemotherapy, surgery combined with radiotherapy and so on.^[3,17]^ In most cases, there was no recurrence or metastasis after complete resection. However, there are also cases of recurrence and metastasis of MNTI.^[3]^ The causes of recurrence may be incomplete surgical excision, implantation of tumor cells during surgery, or multicentric tumors. At present, there is no reliable method to predict the invasiveness of tumors. Since the gross morphological or histological features of MNTI do not indicate its prognosis, early diagnosis and close follow-up after complete resection are essential.

## 4 Conclusion

This case emphasizes that, albeit with extremely low incidence, pediatric surgeons and pathologists must always consider the possibility of MNTI while dealing with ovarian neoplasms in infants. Given its rapid growth potential, early diagnosis is very important to limit local mass expansion and relieve the compression symptoms. Characteristic histopathological features supplemented with immunohistochemistry remain the gold standard for diagnosis. Although considered to be a benign tumor, proper treatment and close clinicoradiological follow-up of this tumor are very important owing to the considerable risk of recurrence.

## Author contributions

**Conceptualization:** Ming Li, Yong Li.

**Resources:** Ming Li, Xianglian Tang, Yaling Xiao, Yong Li.

**Writing – original draft:** Zan Liu.

**Writing – review & editing:** Zan Liu, Xianglian Tang, Yaling Xiao, Zhenghui Xiao, Yong Li.

## References

[1] Rachidi S Sood AJ Patel KG Melanotic neuroectodermal tumor of infancy: a systematic review. J Oral Maxillofac Surg 2015;73:1946–56. 25936939 10.1016/j.joms.2015.03.061

[2] Sharma P Yadav AK Goyal S Melanotic neuroectodermal tumor of infancy: a rare entity. J Oral Maxillofac Pathol 2019;23: Suppl 1: 134–7. 30967743 10.4103/jomfp.JOMFP_237_16 PMC6421908

[3] Kruse-Losler B Gaertner C Burger H Melanotic neuroectodermal tumor of infancy: systematic review of the literature and presentation of a case. Oral Surg Oral Med Oral Pathol Oral Radiol Endod 2006;102:204–16. 16876064 10.1016/j.tripleo.2005.08.010

[4] Borello ED Gorlin RJ Melanotic neuroectodermal tumor of infancy--a neoplasm of neural crese origin. Report of a case associated with high urinary excretion of vanilmandelic acid. Cancer 1966;19:196–206. 4285905 10.1002/1097-0142(196602)19:2<196::aid-cncr2820190210>3.0.co;2-6

[5] Pinheiro TP Carneiro JT Jr de Melo Alves S Jr Melanotic neuroectodermal tumor of infancy in an African-indigenous patient from the Amazon: a case report. Head Face Med 2013;9:35. 24274360 10.1186/1746-160X-9-35 PMC4222608

[6] Kumar Dutta H Jaw and gum tumours in children. Pediatr Surg Int 2009;25:781–4. 19669649 10.1007/s00383-009-2427-6

[7] Kumar A Deepthi V Aggarwal R Melanotic neuroectodermal tumor of infancy: a rare case report. J Oral Maxillofac Pathol 2018;22: Suppl 1: S44–7. 29491604 10.4103/jomfp.JOMFP_197_17 PMC5824516

[8] Chaudhary A Wakhlu A Mittal N Melanotic neuroectodermal tumor of infancy: 2 decades of clinical experience with 18 patients. J Oral Maxillofac Surg 2009;67:47–51. 19070747 10.1016/j.joms.2007.04.027

[9] Barnes DJ Hookway E Athanasou N A germline mutation of CDKN2A and a novel RPLP1-C19MC fusion detected in a rare melanotic neuroectodermal tumor of infancy: a case report. BMC Cancer 2016;16:629. 27519597 10.1186/s12885-016-2669-3 PMC4983003

[10] Fowler DJ Chisholm J Roebuck D Melanotic neuroectodermal tumor of infancy: clinical, radiological, and pathological features. Fetal Pediatr Pathol 2006;25:59–72. 16908456 10.1080/15513810600788715

[11] Allen MS Jr Harrison W Jahrsdoerfer RA Retinal anlage” tumors. Melanotic progonoma, melanotic adamantinoma, pigmented epulis, melanotic neuroectodermal tumor of infancy, benign melanotic tumor of infancy. Am J Clin Pathol 1969;51:309–14. 4304365 10.1093/ajcp/51.3.309

[12] Mirich DR Blaser SI Harwood-Nash DC Melanotic neuroectodermal tumor of infancy: clinical, radiologic, and pathologic findings in five cases. AJNR Am J Neuroradiol 1991;12:689–97. 1652883 PMC8331586

[13] Hameed K Burslem MR A melanotic ovarian neoplasm resembling the “retinal anlage” tumor. Cancer 1970;25:564–7. 4313653 10.1002/1097-0142(197003)25:3<564::aid-cncr2820250310>3.0.co;2-l

[14] Vajtai I Sutak I Varga Z Melanotic progonoma as a component of ovarian teratoma. Histopathology 2000;36:283–5. 10809600 10.1046/j.1365-2559.2000.0872d.x

[15] Gomes CC Diniz MG de Menezes GH BRAFV600E mutation in melanotic neuroectodermal tumor of infancy: toward personalized medicine? Pediatrics 2015;136:e267–9. 26122804 10.1542/peds.2014-3331

[16] Gerald WL Ladanyi M de Alava E Clinical, pathologic, and molecular spectrum of tumors associated with t(11;22)(p13;q12): desmoplastic small round-cell tumor and its variants. J Clin Oncol 1998;16:3028–36. 9738572 10.1200/JCO.1998.16.9.3028

[17] Derache AF Rocourt N Delattre C Melanotic neuroectodermal tumors of infancy: Current state of knowledge. Bull Cancer 2014;101:626–36. 24977451 10.1684/bdc.2014.1985

